# Global trends and Frontier topics about vascular smooth muscle cells phenotype switch: A bibliometric analysis from 1999 to 2021

**DOI:** 10.3389/fphar.2022.1004525

**Published:** 2022-11-14

**Authors:** Ying Han, Langchao Yan, Lu Xia, Shifu Li, Qian Zhang, Chen jin

**Affiliations:** ^1^ Department of Oral and Maxillofacial Surgery, Center of Stomatology, Xiangya Hospital, Central South University, Changsha, China; ^2^ Center for Medical Genetics & Hunan Key Laboratory of Medical Genetics, School of Life Sciences, Central South University, Changsha, China; ^3^ Mini-invasive Neurosurgery and Translational Medical Center, Xi’an Central Hospital, Xi’an Jiaotong University, Xi’an, China; ^4^ Department of Neurosurgery, Xiangya Hospital, Central South University, Changsha, China; ^5^ Hypothalamic-Pituitary Research Center, Xiangya Hospital, Central South University, Changsha, China; ^6^ National Clinical Research Center for Geriatric Disorders, Xiangya Hospital, Central South University, Changsha, China

**Keywords:** vascular smooth muscle cells, phenotype switching, bibliometric, citespace, VOSviewer

## Abstract

**Objective:** Vascular smooth muscle cell phenotype switch (VSMCPS) plays a significant role in vascular remodeling. This study aimed to conduct a bibliometric analysis and visualize the knowledge map of research on VSMCPS.

**Methods:** We retrieved publications focusing on VSMCPS from the Web of Science Core Collection database (SCI-EXPANDED) from 1999 to 2021. Using bibliometric tools, VOSviewer and CiteSpace, we identified the most productive researchers, journals, institutions, and countries. At the same time, the trends, hot topics, and knowledge networks were analyzed and visualized.

**Results:** A total of 2213 publications were included in this analysis. The number of annual publications in the VSMCPS field exhibited an upward trend and could be roughly divided into three phases. Until 2006, the most prolific authors were from the United States. As of 2008, the number of articles published in China increased dramatically to reach 126 papers in 2020. As of 2014, China was the most productive country in this field. The United States ranked first in the number of highly-influential authors, institutions, and literature from 1999 to 2022. Owens GK, Hata, Akiko, and Wen, jin-kun were the most prolific authors. *Arteriosclerosis Thrombosis and Vascular Biology*, *Circulation Research, and Cardiovascular Research* were the top-ranked journals in this field. “Vascular remodeling,” “atherosclerosis,” “neointima,” “hypertension”, and “inflammation” were the main researched topics. New diseases, new mechanisms, and new phenotype (e.g., micro RNA, macrophage-like-cell, hypoxia, autophagy, long noncoding RNA, oxidative stress, endoplasmic reticulum stress, senescence, aging, abdominal aortic aneurysm, and aortic dissection) represent the trending topics in recent years.

**Conclusion:** This study systematically analyzed and visualized the knowledge map of VSMCPS over the past 2 decades. Our findings provide a comprehensive overview for scholars who want to understand current trends and new research frontiers in this area.

## 1 Introduction

Vascular remodeling is an active process of structural alteration involving vascular wall thickening, thinning, and/or lumen stenosis as a result of endothelial injury, vascular smooth muscle cells (VSMCs) proliferation and migration, and extracellular matrix deposition (Galis and Khatri, 2002). It may results in atherosclerosis and/or stenosis, followed by myocardial infarction, stroke, etc. In healthy blood vessels, VSMC interact with the changing hemodynamics and provide structural and functional integrity to the vessel wall. When the endothelium is damaged, VSMCs are exposed to changes in growth factors (PDGF-BB, EGF, TGF, etc.), cytokines (IL-6, CSF, TNF-α, etc.), extracellular matrix (ECM) components, shear stress, oxygen free radicals, and other stimuli (Raines, 2000; Cordes et al., 2009; Vengrenyuk et al., 2015). This phenomenon induces VSMCs decreased expression of contractile proteins and myofilament density, increased expression of ECM components and ECM-remodeling enzymes, meanwhile enhancing the migration and proliferation abilities. This transition from a relatively quiescent contractile phenotype to an active synthetic phenotype is called VSMC phenotype switching (VSMCPS). Current evidence suggests that VSMCPS is involved in the initiation and progression of many cardiovascular and cerebrovascular diseases, such as atherosclerosis, in-stent stenosis, intracranial aneurysms, aortic aneurysms and aortic dissection (Alexander and Owens, 2012; Huang et al., 2020). It is widely thought that VSMCPS plays an important role during the initiation, development, and plaque formation of atherosclerosis (Ikari et al., 1999). At the early stage of atherosclerosis, VSMCs migrated from preexisting medial SMCs or SMC-like-cells derived from circulating blood in neointima tissues exhibit a proliferative and migrative state, decreased contractility and expression of smooth muscle markers, which accelerate the plaque formation (Owens et al., 2004; Shankman et al., 2016; Misra et al., 2018). During the late stages of atherosclerosis, VSMCs can switch to macrophage-like cells, foam cells, mesenchymal stem cell-like cells, extracelluar-matrix-producing cells, and osteochondrogenic cells (Allahverdian et al., 2014; Jacobsen et al., 2017; Basatemur et al., 2019). Eventually, it may result in plaque calcification (Steitz et al., 2001; Naik et al., 2012). Indeed, many biological functions of VSMCs have been characterized, and studies on the mechanism of VSMCPS and new therapeutic approaches targeting this process have significant value. In addition, clarifying the current status and hot topics may help new researchers gain a quick overview of this area for future research design, given the huge volumes of data reported in the literature.

Bibliometric analysis is a big data-based scientific mapping method used to investigate the key contributors (e.g., country, institution, author, and journal) and identify the collaborative network between them in a given field. Additionally, it can identify the main Frontier research topics (Zhang et al., 2022a; Zhang et al., 2022b). Several tools, such as CiteSpace and VOSviewer, are popularly used for bibliometric analysis, primarily to explore the potential relationships between the published literatures and assess progress made in a particular field (Zhang et al., 2022a). Over the years, these two bibliometric tools have been widely used to predict hot topics and future research trends in several medical domains, such as cardiovascular diseases, ARDS, sepsis, host immune response, etc., (Chen, 2006; Avcu et al., 2015; Perianes-Rodriguez et al., 2016; Yao et al., 2020; Ma et al., 2021). To date, no bibliometric analysis of VSMCPS has been reported. Therefore, this study aims to analyze the publication trends and visualize the knowledge network of all publications regarding VSMCPS research. Importantly, this study’s findings can help researchers quickly grasp current knowledge and identify emerging Frontier topics regarding VSMCPS.

## 2 Methods

### 2.1 Data sources

We searched the literature for publications related to VSMCPS from the Web of Science Core Collection database (SCI-EXPANDED). The following search terms and Boolean operators were entered for a database search: TS= (vascular-smooth-muscle-cell* OR vascular SMC* OR VSMC*) AND TS= (phenotyp* transform* OR phenotyp* switch* OR phenotyp* alter* OR phenotyp* chang* OR phenotyp* modulat* OR phenotp* transition OR phenotyp* diversity) AND Article OR Review Article (Document Types) AND English (Languages). The time span was from 1999-01-01 to 2022-09-22.

### 2.2 Bibliometric analysis

After screening by titles and abstracts, we downloaded the retrieved publications as “Full Record and Cited References” and imported the search results into Excel 2010 to analyze the publication trend and major contributors (e.g., authors, institutions, countries, and journals). CiteSpace (Version 6.1. R1) and VOSviewer (version 1.6.11, Leiden University, Leiden, Netherlands) were used to construct the knowledge network, identify the collaborative network, the keyword co-occurrence network, and the co-citation references network. In VOSviewer and CiteSpace, the node size was positively related to the number of publications, and total link strength (TLS) was positively correlated with the cooperation strength.

## 3 Results

### 3.1 General data

**FIGURE 1:**
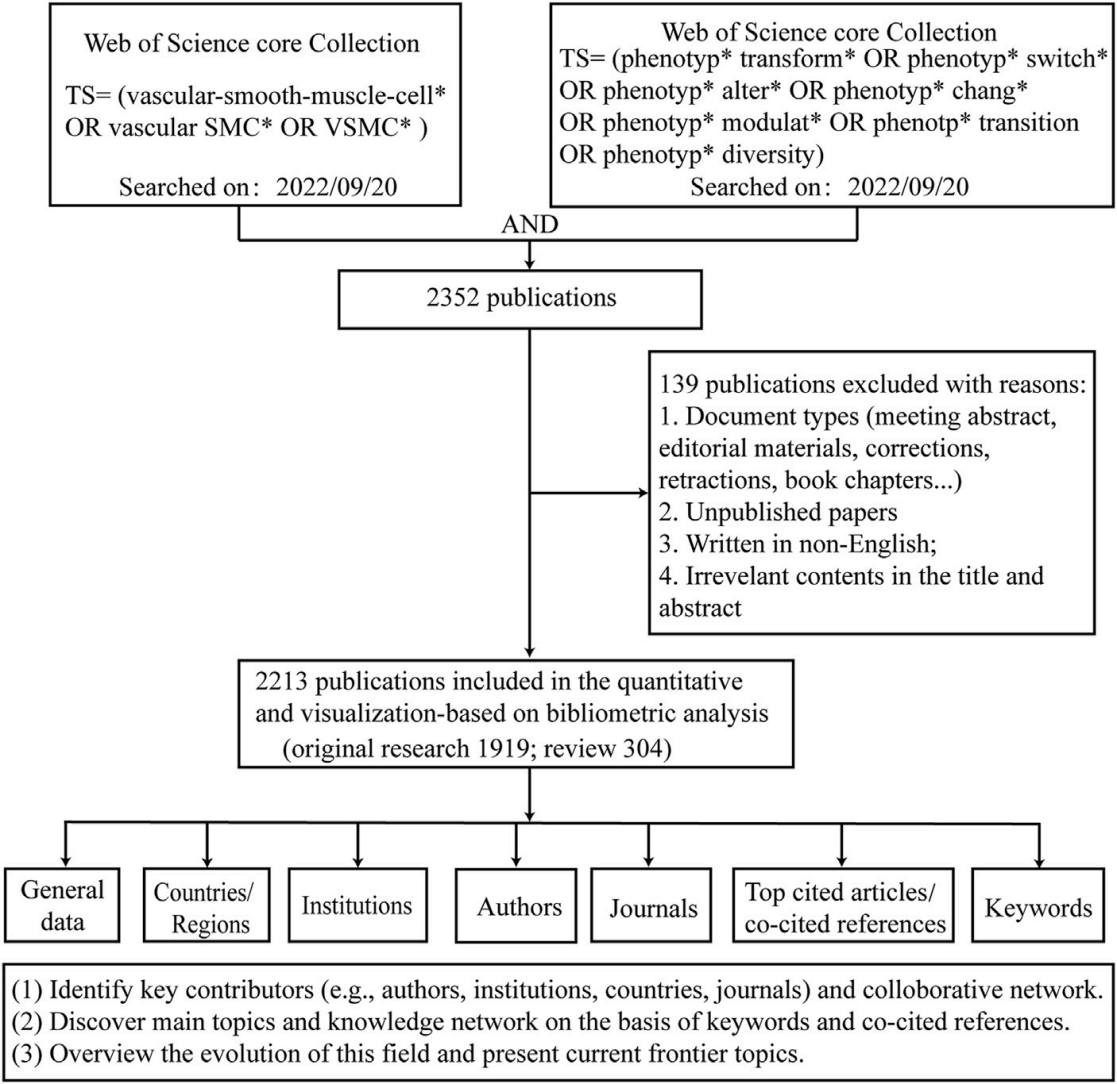
The workflow of data collection and bibliometric analysis.

Figure 1 shows the process of literature screening and bibliometric analysis. A total of 2213 publications were retrieved and imported into bibliometric tools (VOSviewer and CiteSpace) for further analysis. The number of total citations (TC) was 82,487, and the number of citations per publication (CPP) was 37.27. Overall, a total of 65 countries/regions, 1,933 institutions, 11,912 authors, and 579 journals contributed to this field.

### 3.2 Publication trend

**FIGURE 2:**
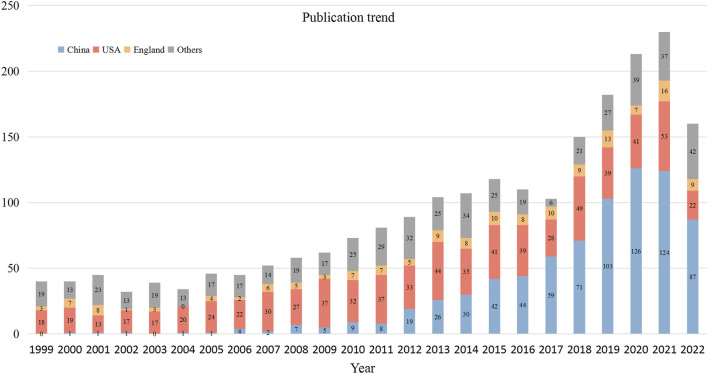
Publication trend of the top three prolific countries/regions.

The number of annual publications in the VSMCPS field exhibited an upward trend and could be roughly divided into three phases. Phase I was from 1999 to 2006, with below 50 publications per year. Phrase II was from 2007 to 2015, and the number of publications steadily increased from 50 to over 100 per year. Phase III was from 2016 to 2022, the number of publications slightly decreased in 2016 and 2017 and increased sharply after 2017 to over 200 publications per year. Figure 2 presents the annual output of the top three contributing countries.

### 3.3 Countries/regions

**FIGURE 3:**
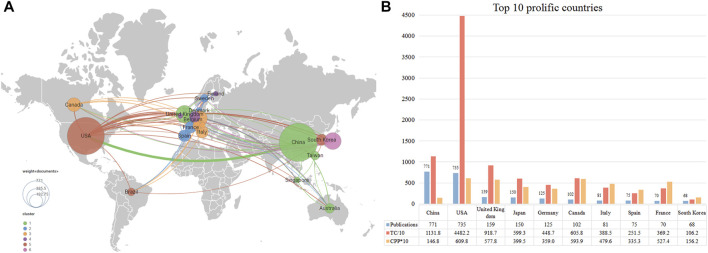
The top 10 most productive countries and international co-operative network. **(A)** Collaboration among prolific countries which published more than 10 papers. Node size represents the number of publication, the width of the link is positively correlated with the strength of cooperation. **(B)** The number of publications, total citations (TC), and citations per publication (CPP) of the top 10 most productive countries/regions.

Figure 3A displays the international collaborative network formed by countries that published at least ten publications in this field. The United States and China were the most significant nodes with the largest number of publications. At the same time, China (TLS = 171) and the United States (348) were actively involved with other countries/regions. Figure 3B lists the top ten contributing countries in this field. China ranked first with 771 publications, followed by the United States (735 publications) and the United Kingdom (159 publications). The top three countries accounted for 75.23% of the total publications. Regarding citations, the United States hold the highest number of TC (*n* = 44,822) and CPP (*n* = 60.98). China ranked second in the number of TC (*n* = 11,318) but ranked 10th in terms of CPP (*n* = 14.68).

### 3.4 Institutions

**FIGURE 4:**
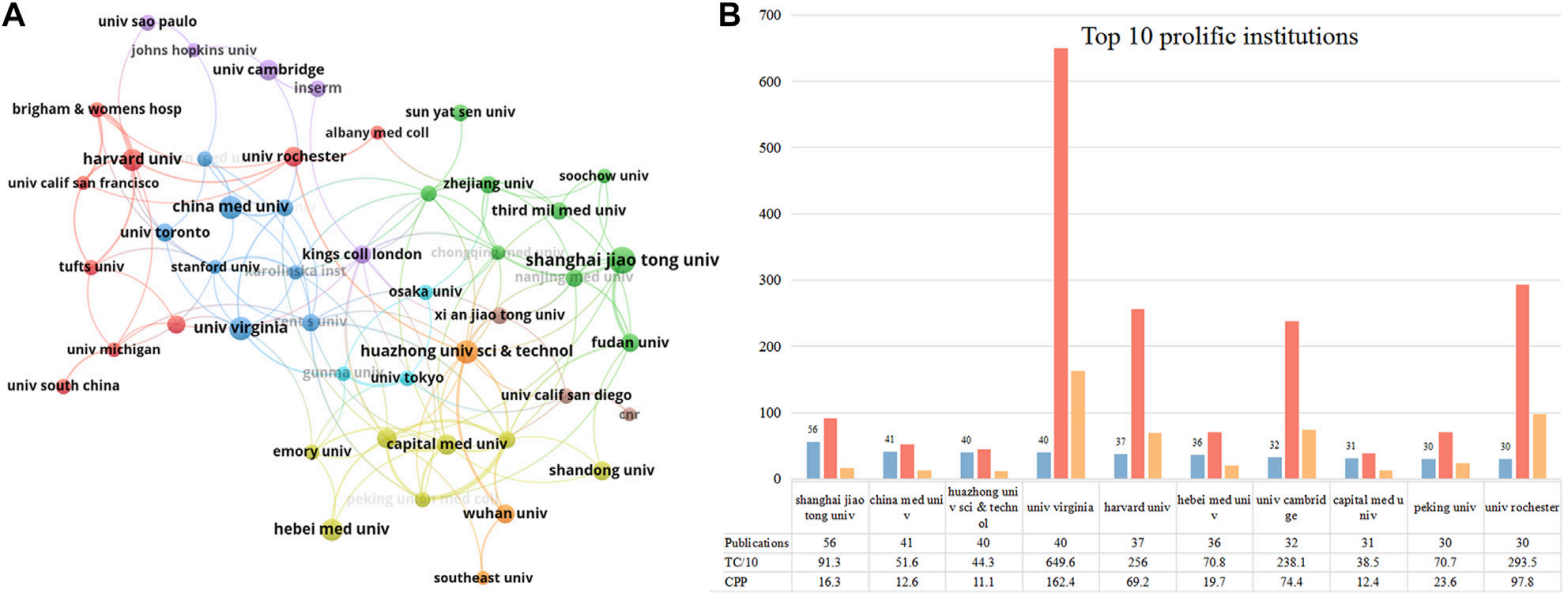
The top 10 most productive institutions and inter-institutions’ co-operative network. **(A)** Collaboration among these prolific institutions which published more than 15 papers. Node size indicates the number of publications. The width of links reflects the cooperative strength. **(B)** The number of publications, total citations (TC), and citations per publication (CPP) in the top 10 prolific institutions.

Figure 4A presents the collaborative network between institutions that published more than 15 publications. Several clusters were formed, suggesting a closed cooperative relationship among them. Institutions in Cluster# red and blue were located in North America (e.g., Harvard Univ, Univ Rochester, and Univ Toronto). Institutions in Cluster# purple were located in Europe (Univ Cambridge, Kings Coll London, and Inserm). Institutions in Cluster# light blue were located in Japan (Osaka Univ, Gunma Univ, and Univ Tokyo). Institutions in Cluster# green, yellow, and brown were located in China (Shanghai Jiaotong Univ, Capital Med Univ, and Heibei Med Univ). We used the timeline view in VOSviewer to visualize the activity of these institutions (Supplementary Figure S1). Institutions in the United States, the United Kingdom, and Japan started relatively early in this domain compared to China. Besides Shanghai Jiaotong Univ and Soochow Univ, other Chinese institutions became active after 2015. Figure 4B lists the top ten contributing institutions in this field. Shanghai Jiaotong Univ (56 publications) ranked first, followed by China Medical Univ (41 publications) and Huazhong Univ Sci&Tec (40 publications). Regarding citations, Univ Virginia scored the highest TC (*n* = 6,496) and CPP (*n* = 162.4). Univ Rochester ranked second with 2,935 TC and 97.8 CPP. Overall, of the top 10 most productive institutions, institutions from China had relatively few citations compared with those from other countries.

### 3.5 Authors

**FIGURE 5:**
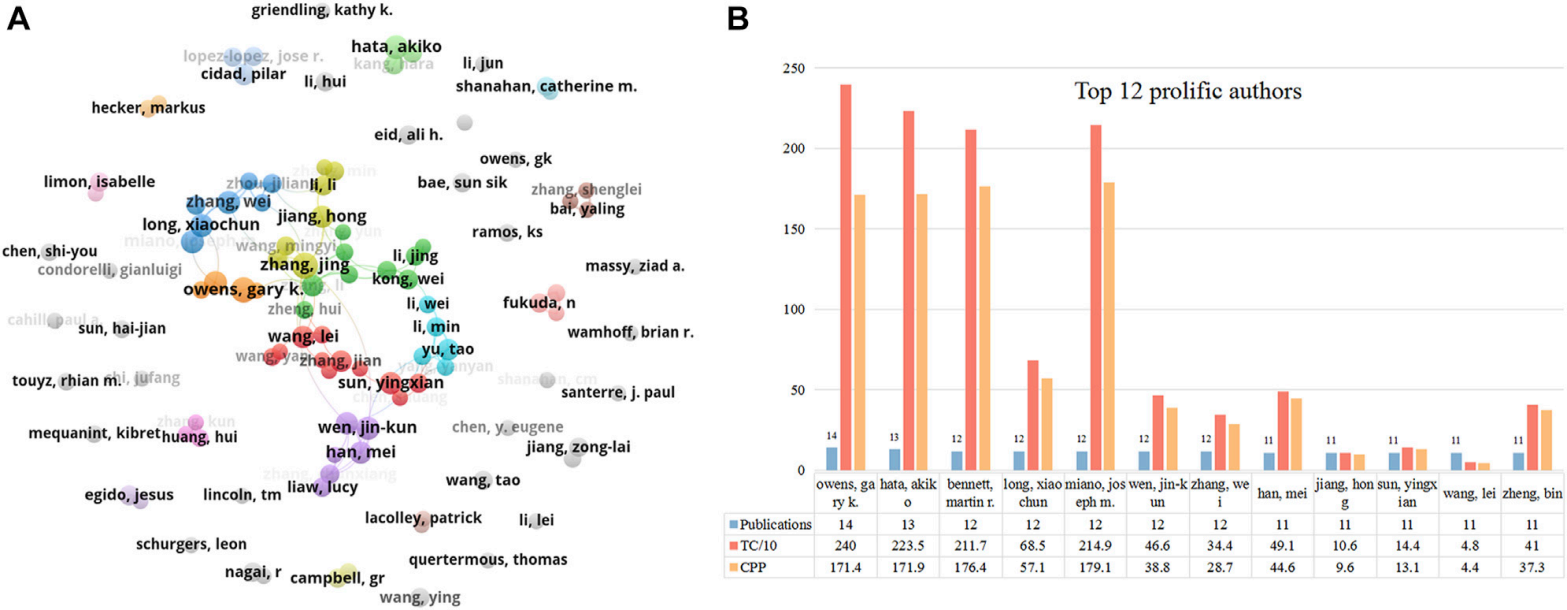
The top 12 most productive authors and collaboration network centered around these authors. **(A)** Collaboration among prolific authors who published more than 6 papers. Node size reflects the number of publications. The width of links represents the cooperative strength. **(B)** The number of publications, total citations (TC), and citations per publication (CPP) in the top 12 prolific authors.

Figure 5A shows the collaborative network between authors who have published over six papers. The biggest cluster consisted of 46 authors, with frequent cooperation among them. Owens GK, Hata, Akiko, and Wen, jin-kun were the central nodes in the cooperative map. Similar to institutions, we used the timeline view in VOSviewer to visualize the activity of these prolific authors (Supplementary Figure S2). Most Chinese scholars were active after 2015 (e.g., Sun Yingxian, Yu Tao, and Zhang Jing). Owens GK, Zhang Wei, Fukuda N, Hata, and Akiko started exploring this field relatively early (around 2010). The top 12 prolific authors who published over ten papers in this field are listed in Figure 5B. Owens GK (University of Virginia, United States) ranked first with 14 papers, followed by Hata Akiko (University of California San Francisco) with 13 papers, and Bennett MR, Long Xiaochun, Miano, JM, Wen jin-kun, and Zhang wei with 12 publications. Regarding citations, Owens GK (2,400 TC, 171.4 CPP), Hata, Akiko (2,235 TC, 171.9 CPP), Bennett MR (2,117 TC, 176.4 CPP), and Miano JM (2,149 TC, 179.1 CPP) were the top four highest citation scholars in this area.

### 3.6 Journals

Table 1 lists the top 10 prolific journals and co-cited journals. *Arteriosclerosis Thrombosis and Vascular Biology (ATVB)* (IF (2021) = 10.5, Q1) was the most prolific journal, with 100 publications (4.5% of the total), followed by *Circulation Research* (IF (2021) = 23.2, Q1) with 82 publications, and *Cardiovascular Research* (IF (2021) = 13.1, Q1) with 55 publications. In terms of citations, *Circulation Research* ranked first (10,169 TC, 124 CPP), followed by *ATVB* (4,977 TC, 49.8 CPP) and *Cardiovascular Research* (4,125 TC, 75 CPP). However, it should be borne in mind that the number of publications may not represent its influence on a given field. Therefore, we used VOSviewer to identify the co-cited journals that frequently cited in the VSMCPS field. The top three co-cited journals were *Circulation Research* (7,444 co-citations), *Journal of Biological Chemistry* (5,741 co-citations), and *ATVB* (5,737 co-citations).

**TABLE 1:** Top 10 prolific journals and co-cited journals.

Rank	Journal	Publications	TC	CPP	If (2021)	Co-cited journal	Co-citations	If (2021)
1	Arteriosclerosis Thrombosis and Vascular Biology	100	4977	49.8	10.5	Circulation Research	7444	23.2
2	Circulation Research	82	10169	124.0	23.2	Journal of Biological Chemistry	5741	5.5
3	Cardiovascular Research	55	4125	75.0	13.1	Arteriosclerosis Thrombosis and Vascular Biology	5737	10.5
4	Journal of Biological Chemistry	55	3329	60.5	5.5	Circulation	4030	39.9
5	Atherosclerosis	48	1493	31.1	6.8	PNAS	2569	12.8
6	Plos One	48	1609	33.5	3.8	Cardiovascular Research	2445	13.1
7	Biochemical and Biophysical Research Communications	43	943	21.9	3.3	Journal of Clinical Investigation	2307	19.5
8	International Journal of Molecular Sciences	35	473	13.5	6.2	Nature	2152	69.5
9	Scientific Reports	31	454	14.6	5.0	Cell	1577	66.9
10	Journal of Cellular Physiology	30	994	33.1	6.5	American Journal of Physiology-Heart and Circulatory Physiology	1541	5.1

Abbreviations: TC, total citations; CPP, citations per publication; IF, impact factors.

### 3.7 Top cited articles and co-cited references

**FIGURE 6:**
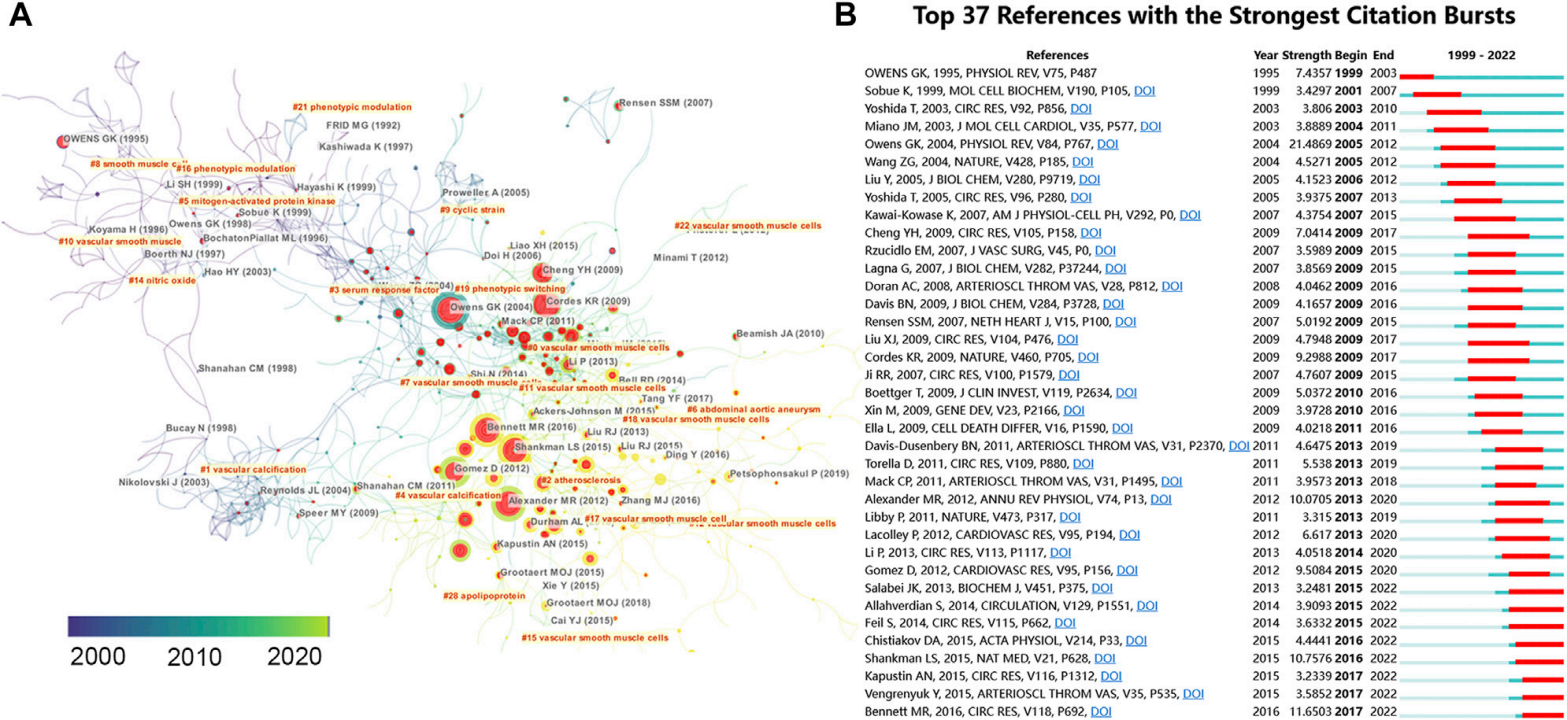
**(A)** Reference co-citation network identified by CiteSpace. The nodes and links are distinguished by colors, in which blue reflects an earlier co-citation relationship, yellow represents a recent co-citation relationship. The size of nodes is positively associated with the citation number. The red nodes represent the publication has burst change which may connect authors in different sub-domains in a given field. **(B)** The top 37 co-cited references with the strongest citation bursts represent important publications in different periods. The red bar indicates the burst duration. The burst strength suggests the importance to the research field.

To identify the most influential studies in this field, we extracted the top 10 most cited and most co-cited publications using VOSviewer and CiteSpace. Table 2 lists the top 10 most cited publications regarding VSMCPS, including five original articles and five reviews. The most cited paper (2,384 TC) was produced by Owens et al. (Owens et al., 2004) published in Physiological Reviews in 2004, entitled “Molecular regulation of vascular smooth muscle cell differentiation in development and disease”. In this review, the authors summarized the mechanisms that control the differentiated state of the VSMCs in normal conditions and how these regulatory processes are altered during the development of intimal lesions in atherosclerosis or vascular injury. Additionally, three publications reported on the function of microRNA in VSMCs (Davis et al., 2008; Cheng et al., 2009; Cordes et al., 2009) and four on the role of VSMCs in atherosclerosis (Jono et al., 2000; Johnson et al., 2006; Bennett et al., 2016). Co-cited references are publications co-cited by at least two articles in a given field (Chen, 2006). Citation burst in CiteSpace was used to detect co-cited references that received relatively more attention from researchers during a specific time. Thirty seven co-cited references (more than 60 co-citations) were identified in Figures 6A,B, which showed the knowledge network evolution in this field. Papers that received more attention in the early years were colored dark blue, and publications primarily gained attention in the past few years were colored yellow. The publications with highest citation burst (*n* = 21.48) was Owens GK, 2004, PHYSIOL REV, V84, P767 (Owens et al., 2004). Publications with citation bursts ending in 2021 were as follows: Salabei JK, 2013, BIOCHEM J, V451, P375 (Salabei et al., 2013); Allahverdian S, 2014, CIRCULATION, V129, P1551 (Allahverdian et al., 2014); Feil S, 2014, CIRC RES, V115, P662 (Feil et al., 2014); Chistiakov DA, 2015, ACTA PHYSIOL, V214, P33 (Chistiakov et al., 2015); Shankman LS, 2015, NAT MED, V21, P628 (Shankman et al., 2015); Kapustin AN, 2015, CIRC RES, V116, P1312 (Kapustin et al., 2015); Vengrenyuk Y, 2015, ARTERIOSCL THROM VAS, V35, P535 (Vengrenyuk et al., 2015); Bennett MR, 2016, CIRC RES, V118, P692 (Bennett et al., 2016).

**TABLE 2:** Top 10 cited publications regarding VSMCPS.

Rank	Authors	Title	Document type	TC	Since 2013 usage count	Journal	Publication year
1	Owens, GK	Molecular regulation of vascular smooth muscle cell differentiation in development and disease	Review	2384	241	Physiol Rev	2004
2	Cordes, KR	miR-145 and miR-143 regulate smooth muscle cell fate and plasticity	Article	1201	132	Nature	2009
3	Jono, S	Phosphate regulation of vascular smooth muscle cell calcification	Article	1108	42	Circ Res	2000
4	Davis, BN	SMAD proteins control DROSHA-mediated microRNA maturation	Article	1039	98	Nature	2008
5	Griendling, KK	Modulation of protein kinase activity and gene expression by reactive oxygen species and their role in vascular physiology and pathophysiology	Review	753	34	ATVB	2000
6	Bennett, MR	Vascular Smooth Muscle Cells in Atherosclerosis	Article	751	186	Circ Res	2016
7	Johnson, RC	Vascular calcification - Pathobiological mechanisms and clinical implications	Review	674	98	Circ Res	2006
8	Rensen, SSM	Regulation and characteristics of vascular smooth muscle cell phenotypic diversity	Review	579	80	Neth Heart J	2007
9	Basatemur, G	Vascular smooth muscle cells in atherosclerosis	Review	564	228	Nat Rev Cardiol	2019
10	Cheng, YH	MicroRNA-145, a Novel Smooth Muscle Cell Phenotypic Marker and Modulator, Controls Vascular Neointimal Lesion Formation	Article	537	48	Circ Res	2009

### 3.9 Keywords’ evolution

**FIGURE 7:**
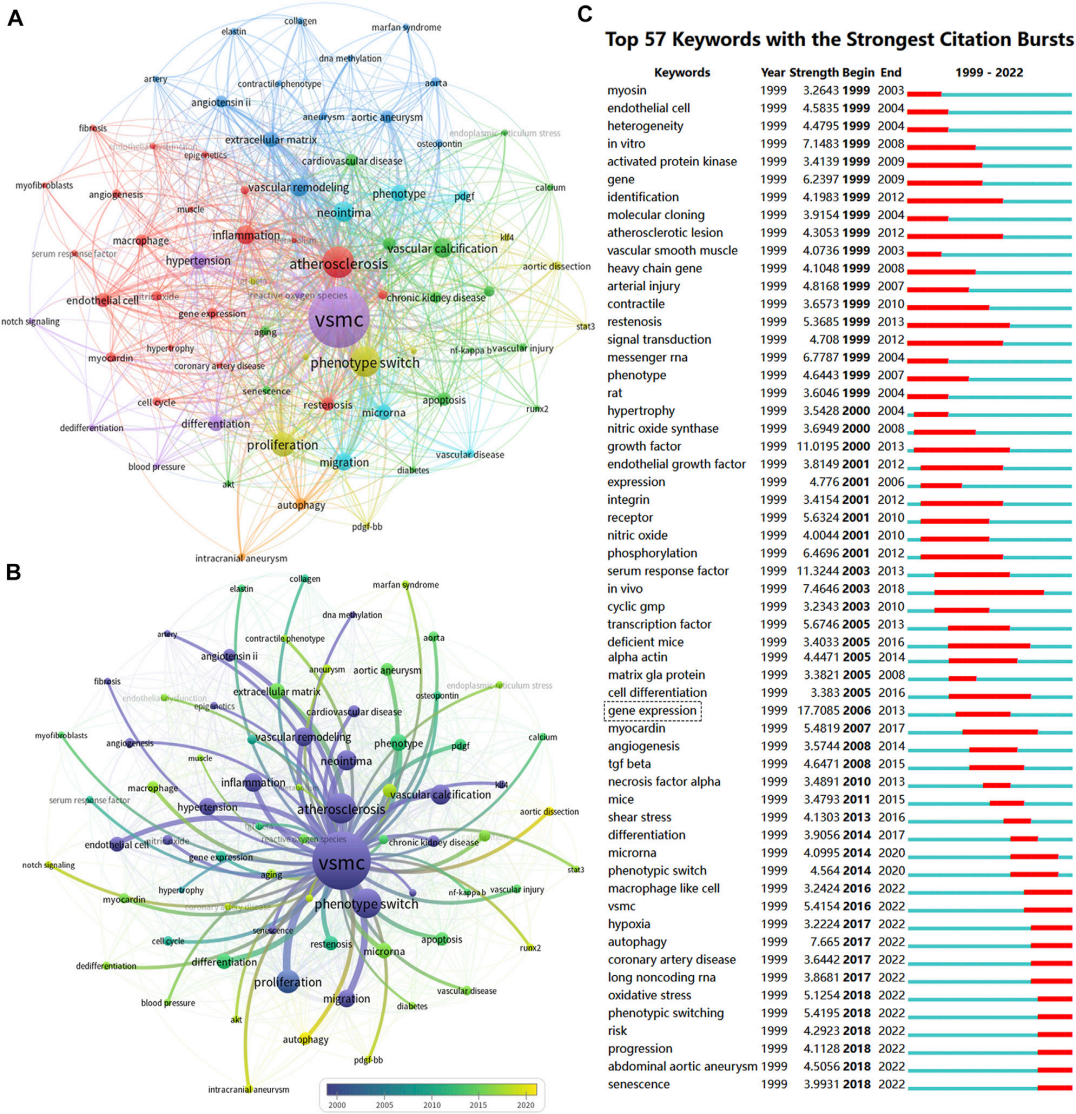
Co-occurrence analysis of keywords. **(A)** The co-occurrence networks of author keywords were visualized by VOSviewer. Large nodes represent keywords with relative higher occurrence; Same color indicates relatively closer relationship; **(B)** The timeline view of keywords in VOSviewer, blue indicates occurred frequently in early stage (around 2000), yellow indicates occurred frequently in late stage (around 2020). **(C)** The top 57 keywords with the strongest citation bursts represent hot topics in different periods. The red bar indicates the burst duration. The burst strength refers to the importance to the research field.

Keyword co-occurrence analysis (VOSviewer) and keyword burst detection (CiteSpace) were used to identify research hotspots shifting and emerging topics. Keywords with similar meanings were merged, such as vascular smooth muscle cell, and vascular SMC were replaced by VSMC (Supplementary Table S1). Closely-related keywords were automatically identified by VOSviewer and labeled the same color. Nodes with larger sizes indicate keywords that occurred more frequently; lines with wider widths suggested they were linked closely together. As shown in Figure 7A, 73 keywords that appeared more than 10 times were grouped into six clusters. The top 10 keywords frequently used by scholars were “VSMC” (*n* = 1003), “atherosclerosis” (*n* = 268), “phenotype switch” (*n* = 246), “proliferation” (*n* = 158), “neointima” (*n* = 114), “vascular calcification” (*n* = 111), “inflammation” (*n* = 100), “vascular remodeling” (*n* = 96), “migration” (*n* = 86), “hypertension” (*n* = 80). Moreover, we used the timeline view of keywords to visualize the evolution of keywords in chronological order. In Figure 7B, keywords (e.g., vascular remodeling, vascular calcification, atherosclerosis, inflammation, and neointima) in dark blue implied that these topics were researched relatively early in this field. In contrast, keywords colored in yellow-green (e.g., apoptosis, aortic aneurysm, restenosis, extracellular matrix, vascular injury, microRNA, reactive oxygen species, oxidative stress, autophagy, aortic dissection, intracranial aneurysm, aging, runx2, endoplasmic reticulum stress, macrophage) indicated that these topics gained more attention in recent years. Figure 7C lists the top 57 keywords with the strongest citation bursts in the field of VSMCPS. Red and blue represent active and inactive periods, respectively. Since 2014, keywords such as “micro RNA, macrophage-like-cell, hypoxia, autophagy, coronary artery disease, long noncoding RNA, oxidative stress, senescence, abdominal aortic aneurysm” were frequently used.

## 4 Discussion

In the era of big data, new researchers can quickly get insights into a particular research field through bibliometric analysis. However, no study has hitherto provided a comprehensive overview of research on VSMCPS, which is significant in understanding the pathogenesis of cardiovascular diseases. Therefore, in this study, we retrieved publications related to VSMCPS and identified the main contributors in this field. In addition, we visualized the evolution of hot topics in this field over the past 2 decades.

### 4.1 Current status and major contributing countries

The output of publications reflects the interest of researchers in a field (Durieux and Gevenois, 2010). Overall, the number of publications in this field exhibited an upward trend. We found that until 2006, the number of publications was mostly less than 50. During this period, the most prolific authors were from the United States, with 20–30 publications (accounting for almost 50% of the total) per year before 2008. Since then, the number of publications from China has increased dramatically, from less than 10 before 2011 to 126 papers in 2020. In contrast, the annual publications from the United States stabled at 30 to 40 during this period. As of 2014, China became the most productive country in this field, as observed in the timeline view map of prolific authors and institutions (Supplementary Figure S1,2), where authors and institutions from the United States are colored dark blue, suggesting they were active relatively early in this field. Authors and institutions from China are colored yellow-green, indicating they were active in recent years. Moreover, the co-cited references analysis and co-occurrence keyword analysis showed that the frequency of keyword “gene expression” increased sharply after 2006 (the highest citation burst keyword with 17.7), and references with strong citation bursts were blossomed after 2007. This finding indicated that the VSMCPS field has flourished since 2007, which was validated in the publication trend map (Figure 2).

### 4.2 Active institutions and authors

To identify the most influential research teams, we used VOSviewer to identify the active institutions and authors, which may help scholars to choose potential partners in the VSMCPS field. Owens GK (University of Virginia, United States) was the most prolific and cited author. He and his colleagues summarized the mechanisms and regulatory processes of VSMCs in normal conditions and the formation of intimal lesions in atherosclerosis or vascular injury (Owens et al., 2004) in 2004. This study was the most cited paper and was the publication with the strongest citation burst (Table 2; Figure 6). Subsequently, they comprehensively reviewed and updated the significant role of VSMCs in various vascular diseases, including aortic aneurysm (Ailawadi et al., 2009), cerebral aneurysm (Starke et al., 2014) and atherosclerosis (Alexander and Owens, 2012; Bennett et al., 2016). New researchers should follow Owens GK and read these publications to obtain a theoretical basis in VSMCPS field. We also identified the most productive authors and institutions. There were six Chinese institutions, three United States institutions and one British institution in the top 10 prolific institutions. Moreover, there were seven active Chinese scholars (Wen jinkun, Zhang wei, etc.), four active United States scholars (Owens GK, Hata Akiko, Long Xiaochun, Miano, JM) and one British author (Bennett MR) in the top 12 most productive authors. However, United States and British institutions and authors were associated with higher citations (TC and CPP) than Chinese ones. Several factors may explain China’s contradiction between the quantity and quality of publications. First, compared with United States and British scholars, Chinese scholars explored this domain relatively late. In fact, China surpassed the United States in the number of publications for the first time in 2015, suggesting that Chinese publications may need more time to get cited. Besides, we found that the percentage of publications in *Circulation Research, ATVB*, and *Cardiovascular Research* (the highest citation journal) by authors from the United States and the United Kingdom were higher than their Chinese counterparts (Supplementary Table S2). Indeed, if Chinese scholars want to improve their influence in this field, they may deepen their research and choose more influential journals for their manuscript submission in the future.

### 4.3 Active journals and co-cited journals

Knowledge of the most prolific journals is essential to help researchers before submitting manuscripts, and top co-cited journals could be used as authoritative journals in a given field. *ATVB* was the most prolific journal with 100 publications, including 97 original research studies and seven reviews. *Circulation Research* was the second most prolific journal, including 75 original research studies and 7 reviews) with many citations (10,169 TC and 12.4 CPP). *Cardiovascular Research* ranked third with 55 publications (37 original research studies and 18 reviews), 4125 TC, and 75 CPP. To some extent, the relatively higher proportion of reviews in *Circulation Research* and *Cardiovascular Research* explained their greater number of citations.

### 4.4 Research hotspots and Frontier trend

The keyword co-occurrence analysis and co-cited references were used to visualize the knowledge network and Frontier research in a field (Chen, 2004). Over the past 20 years, the top ten frequently used keywords were “VSMC,” “atherosclerosis,” “phenotype switch,” “proliferation,” “neointima,” “vascular calcification,” “inflammation,” “vascular remodeling,” “migration,” “hypertension,” indicating that the mechanism of VSMCPS in vascular remodeling diseases (e.g., atherosclerosis, restenosis, and hypertension) was a major research hotspot.

We used the timeline view of VOSviewer and the keyword burst of CiteSpace to visualize the shifting of hot topics (Figures 7B,C). The research hotspots shifted from topics (e.g., vascular injury and restenosis, *in vivo* and *in vitro* models, activation, transcription, inhibition, proliferation, apoptosis, gene expression, differentiation, and atherosclerosis) to topics regarding inflammation, macrophage, oxidative stress, autophagy, endoplasmic reticulum stress, aging, intracranial aneurysm, microRNA, and aortic dissection. CiteSpace’s keyword burst analysis also yielded similar keyword shifts. Senescence, autophagy, oxidative stress, microRNA, macrophage-like-cell, and abdominal aortic aneurysms gained more attention in recent years. These new keywords are further discussed in the following section.

#### 4.4.1 Emerging diseases

Over the years, many researchers have explored the function of VSMCs in common cardiovascular diseases, such as atherosclerosis (Bennett et al., 2016; Basatemur et al., 2019), hypertension (Lacolley et al., 2012; Touyz et al., 2018), in-stent stenosis (Wu et al., 2019). These diseases are characterized by artery injury, and thus arterial stiffness, neointimal formation, or stenosis. Corresponding drugs have been developed and used in clinics globally, including statins, anti-hypertensive drugs, and sirolimus. However, intracranial aneurysm, aortic dissection, aortic aneurysm, and Marfan syndrome, characterized arterial dilatation, often have poor prognoses due to the lack of efficient therapeutic drugs. Accordingly, researchers have focused on these diseases in recent years. Owens GK and his colleagues demonstrated that TNF-α could promote VSMCs pro-inflammatory/matrix-remodeling phenotype and accelerate the formation of intracranial aneurysms. Treatment with a TNF-α inhibitor *in vivo* could reverse aneurysmal change during the formation of intracranial aneurysms (Ali et al., 2013). Importantly, they summarized the function of VSMCs in intracranial aneurysm pathogenesis in 2014 and pointed out that targeting VSMCPS may retard the progression of intracranial aneurysm (Starke et al., 2014). Similarly, Rombouts et al. reviewed the function of VSMCs in aortic aneurysm and aortic dissection and pointed out that TGF-β signaling and its’ regulatory RNA expression could modulate VSMCPS, and maybe potential targets for noninvasive AA and AD treatment options (Michel et al., 2018; Rombouts et al., 2022).

#### 4.4.2 New phenotypes and mechanisms

The historical view of VSMCPS is that relatively quiescent VSMCs acquire the capability of hyper-proliferation and hyper-migration when spurred by various stimuli. However, the mechanisms underlying VSMCPS are largely unknown. Recently, researchers have shifted their focus to emerging phenotypes and new mechanisms. For example, Grootaert et al. reviewed the function of VSMCs in advanced atherosclerotic plaques and pointed out that defective VSMCs autophagy could accelerate the development of stress-induced premature senescence and atherogenesis (Grootaert et al., 2018). Starke et al. reported that cigarette smoke exposure initiated oxidative stress-induced VSMCPS and the formation and rupture of intracranial aneurysm (Starke et al., 2018). Lu et al. (2021) summarized that the loss of VSMCs, VSMCPS, elevated reactive oxygen species, defective autophagy, and increased senescence could contribute to aortic aneurysm development. Additionally, non-coding RNA (e.g., microRNA, LncRNA, and cirRNA) are also received much attention from researchers. Cordes et al. first demonstrated that miR-143 and miR-145 are cardiac and SMC-specific microRNAs. MiR-145 can control VSMCs fate, and miR-145 and miR-143 co-function to regulate VSMCPS. Song et al. (2018) reported that LncRNA MALAT1 regulates VSMCPS *via* activation of autophagy. Wang et al. reported that Hsa_circ_0031608 might be involved in the rupture of intracranial aneurysm *via* regulating VSMCPS (Wang et al., 2022). Arencibia et al. showed that lncRNAs regulate VSMCPS and participate in the pathological process of in-stent stenosis (Arencibia et al., 2022). Additionally, He et al. showed that circChordc1 could suppress vascular remodeling and reverse pathological aortic aneurysm progression through optimized VSMCPS and improved their growth (He et al., 2022). However, hitherto, no effective drugs targeting these non-coding RNA are available for clinical practice.

#### 4.2.3 Cell-cell interactions

Macrophages and their interactions with VSMCs have become research hotspots in recent years. Pro-inflammatory (M1) and anti-inflammatory (M2) macrophages have been documented in various vascular diseases (Chen et al., 2020; Zhao et al., 2021). Barrettet al. reported that inflammation caused by the imbalance of M1/M2 might be a major factor affecting the VSMCPS and thus atherosclerosis plaque stability (Moore et al., 2013; Barrett, 2020). Moreover, VSMCs can switch to macrophage-like cells after cholesterol loading, and this alteration may play an important role in atherosclerosis advancement (Liu et al., 2017). In aneurysms, macrophages can infiltrate the aneurysm wall, secrete pro-inflammation factors, promote VSMCs apoptosis and death, weaken the vascular wall, and lead to the progression and rupture of the aneurysm (Hadi et al., 2018; Frosen et al., 2019). A study found that knock out of macrophage matrix metalloproteinases-9 can mitigate macrophage infiltration in blood vessels, reduce the abundance of inflammatory factors, and retard the formation and rupture of aneurysms (Longo et al., 2002). Additionally, VSMCs in neointima lesions could promote the maturation of macrophages and activate VSMCPS. Both processes can accelerate the progression of arterial stenosis after injury (Ostriker et al., 2014). However, the detailed crosstalk between macrophage and VSMCs remain to elucidate in the future.

#### 4.2.4 New techniques

Recent single-cell sequencing and lineage tracing works have shown that VSMCs are more diverse than their previously recognized contractile or proliferative/synthetic phenotypes. VSMCs can adopt alternative phenotypes, including those resembling foam cells, macrophages-like cells, mesenchymal stem cells and osteochondrogenic cells, which contribute positively and negatively to disease progression (Basatemur et al., 2019). Tang et al. conducted a lineage tracing with smooth muscle myosin heavy chain (as a marker) and found that in response to vascular injuries, multipotent vascular stem cells, instead of mature VSMCs, become proliferative and differentiate into VSMCs and chondrogenic cells, thus participating in vascular remodeling and neointimal formation (Tang et al., 2012). Using multicolor lineage labeling, Chappell et al. demonstrated that a low proportion of highly proliferative and plastic VSMCs rather than migration from nearby VSMCs could result in injury-induced neointimal lesions and progression of atherosclerotic plaques. Thus, further therapies could be developed targeting these hyperproliferating VSMCs without affecting vascular integrity to reduce vascular diseases (Chappell et al., 2016). Dobnikar et al. (2018) combined single-cell sequencing with lineage tracing to examine VSMCs heterogeneity in healthy mouse vessels. They found that a rare population of VSMC-lineage cells which express Sca1 (the multipotent progenitor marker), suggesting local VSMCs also have the ability of multilineage differentiation. It is worth exploring whether these markers could also be used as disease-relevant transcriptional signatures in VSMC-lineage cells, and be used for disease susceptibility, diagnosis and prevention in the future.

## 5 Limitations

Several limitations were found in this study due to the inherent nature of any bibliometric analysis. First, since the database updates, there may be incomplete literature retrieval, which may cause selection bias. Second, we only selected the Web of Science core collection-EXPANDED database as our data source. Indeed other databases, such as PubMed, Scopus, and Google Scholar, could also be used to increase the robustness of our findings. Third, only CPP and TC were used to measure the academic influence. G-index, H-index, SJR, CiteScore, and SNIP are also indicators of the quality of publications or journals. Fourth, our study only included English literature and excluded non-English publications. Finally, this study did not analyze or compare the funding of different studies.

## 6 Conclusion

This study analyzed and visualized the current status and research trends in the field of VSMCPS using VOSviewer and CiteSpace. Future research directions in this field include VSMCs function in dilated vascular diseases (aneurysm and aortic dissection), exploration of new mechanisms, new phenotypes and cell-cell interaction (e.g., micro RNA, macrophage-like-cell, hypoxia, autophagy, lncRNA, oxidative stress, endoplasmic reticulum stress, senescence, aging) and the implementation of new techniques (single-cell sequencing and lineage tracing) for studying VSMCs function in various vascular diseases.

## Data Availability

The original contributions presented in the study are included in the article/Supplementary Material, further inquiries can be directed to the corresponding authors.
