# Supplementary figures and images for: Global trends and Frontier topics about vascular smooth muscle cells phenotype switch: A bibliometric analysis from 1999 to 2021
Source: Front Pharmacol. 2022 Nov 14;13:1004525. doi: 10.3389/fphar.2022.1004525 (PMC9702355; doi:10.3389/fphar.2022.1004525)

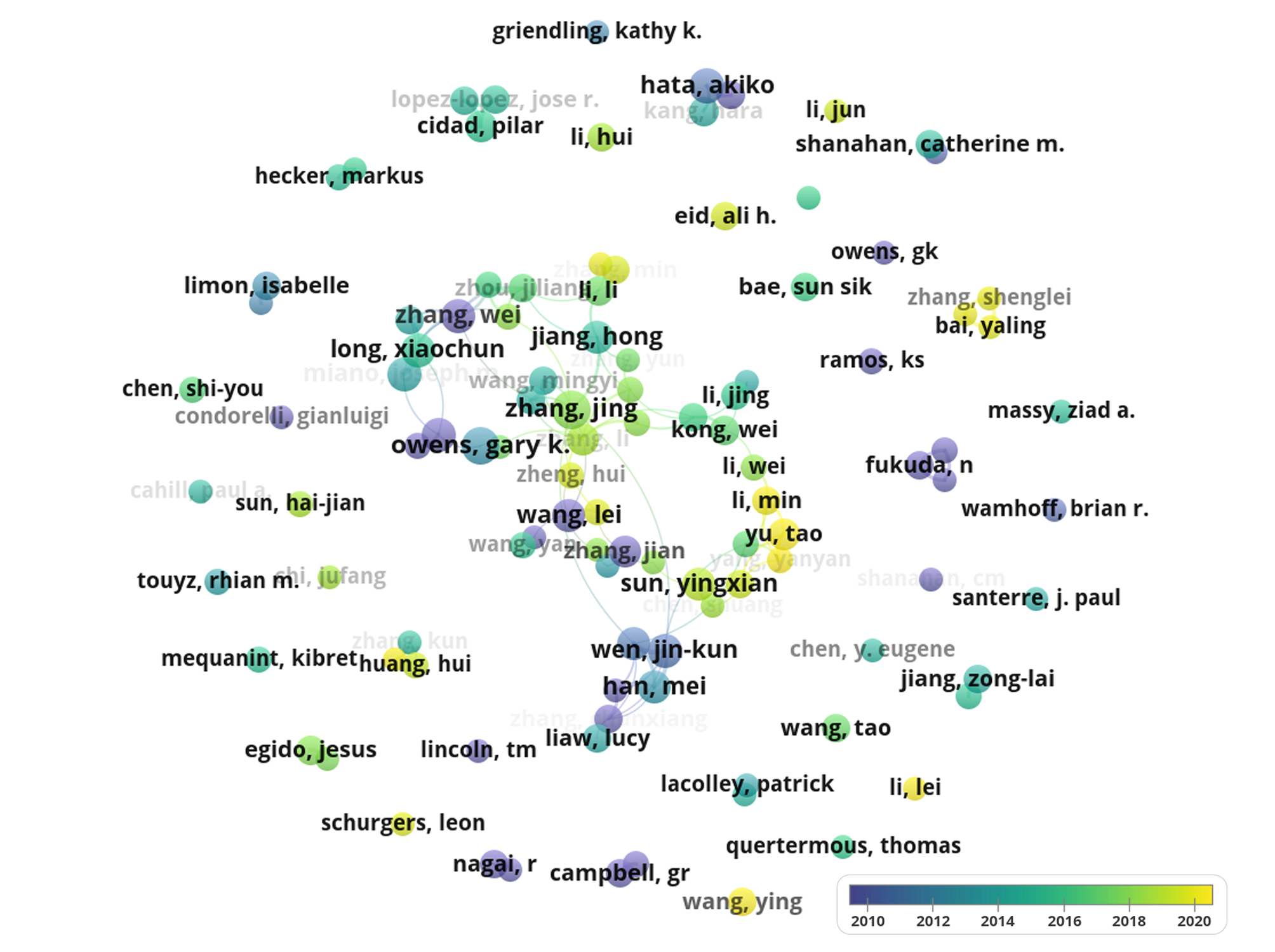

Supplement: Supplementary file 1 [file Image2.TIF]

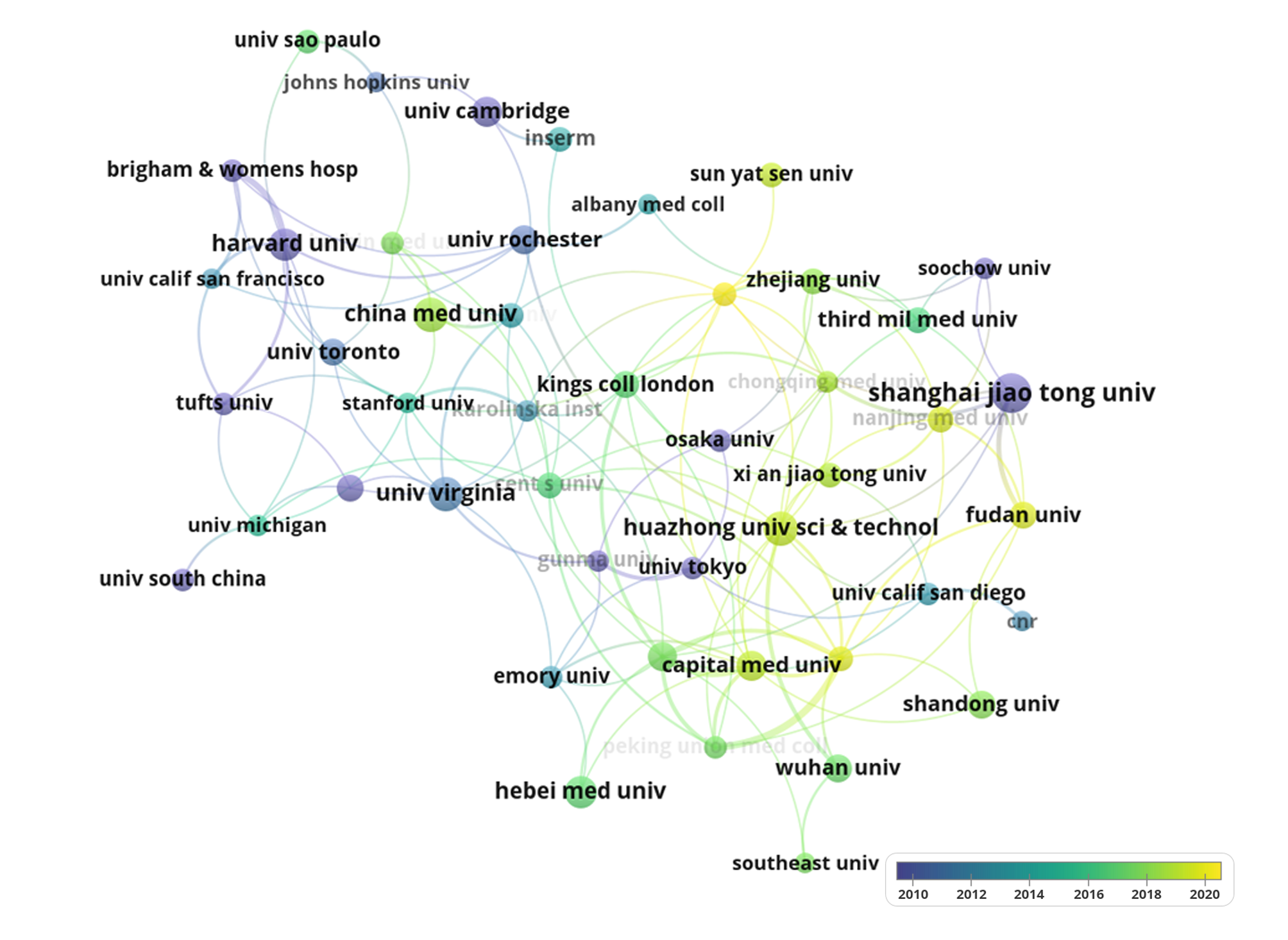

Supplement: Supplementary file 2 [file Image1.TIF]
