# Supplementary material for: Global trends and Frontier topics about vascular smooth muscle cells phenotype switch: A bibliometric analysis from 1999 to 2021
Source: Front Pharmacol. 2022 Nov 14;13:1004525. doi: 10.3389/fphar.2022.1004525 (PMC9702355; doi:10.3389/fphar.2022.1004525)
Supplement: Supplementary file 3 [file Table3.DOCX]

Table S1. Thesaurus

| label | replace by |
| --- | --- |
| smooth muscle cells | VSMC |
| smooth-muscle-cells | VSMC |
| vascular smooth muscle cells | VSMC |
| vascular smooth muscle cell | VSMC |
| smooth muscle cells | VSMC |
| VSMC | VSMC |
| vsmcs | VSMC |
| vascular smooth muscle | VSMC |
| smooth muscle cell | VSMC |
| vascular smooth muscle cells (VSMCS) | VSMC |
| smooth muscle | VSMC |
| endothelium | endothelial cell |
| endothelial cells | endothelial cell |
| micrornas | microrna |
| neointima formation | neointima |
| neointimal formation | neointima |
| intimal hyperplasia | neointima |
| phenotypic switching | phenotype switch |
| phenotypic modulation | phenotype switch |
| neointimal hyperplasia | neointima |
| phenotype switching | phenotype switch |
| phenotypic switch | phenotype switch |
| phenotypic transition | phenotype switch |
| phenotypic transformation | phenotype switch |
| phenotype transformation | phenotype switch |
| phenotype modulation | phenotype switch |
| macrophages | macrophage |
| cardiovascular diseases | cardiovascular disease |
| remodeling | vascular remodeling |
| platelet-derived growth factor | pdgf |
| cell proliferation | proliferation |
| cell migration | migration |
| cell differentiation | differentiation |
| abdominal aortic aneurysm | aortic aneurysm |
