# Supplementary material for: Global trends and Frontier topics about vascular smooth muscle cells phenotype switch: A bibliometric analysis from 1999 to 2021
Source: Front Pharmacol. 2022 Nov 14;13:1004525. doi: 10.3389/fphar.2022.1004525 (PMC9702355; doi:10.3389/fphar.2022.1004525)
Supplement: Supplementary file 4 [file Table4.DOCX]

Table S2. Distribution of journal submissions in the top three prolific countries

|  | Journal | Publications | Percent (%) |
| --- | --- | --- | --- |
| China |  | | |
| 1 | Biochemical and Biophysical Research Communications | 22 | 2.853 |
| 2 | Molecular Medicine Reports | 19 | 2.464 |
| 3 | Arteriosclerosis Thrombosis and Vascular Biology | 17 | 2.205 |
| 4 | Atherosclerosis | 15 | 1.946 |
| 5 | Journal of Cellular and Molecular Medicine | 15 | 1.946 |
| 6 | Experimental and Therapeutic Medicine | 14 | 1.816 |
| 7 | Life Sciences | 14 | 1.816 |
| 8 | Circulation Research | 13 | 1.686 |
| 9 | Frontiers In Cardiovascular Medicine | 13 | 1.686 |
| 10 | Journal of The American Heart Association | 13 | 1.686 |
| USA |  | | |
| 1 | Circulation Research | 53 | 7.191 |
| 2 | Arteriosclerosis Thrombosis and Vascular Biology | 52 | 7.056 |
| 3 | Journal of Biological Chemistry | 33 | 4.478 |
| 4 | Plos One | 29 | 3.935 |
| 5 | American Journal of Physiology Heart and Circulatory Physiology | 20 | 2.714 |
| 6 | Cardiovascular Research | 19 | 2.578 |
| 7 | American Journal of Physiology Cell Physiology | 17 | 2.307 |
| 8 | Circulation | 13 | 1.764 |
| 9 | Journal of The American Heart Association | 13 | 1.764 |
| 10 | Journal of Cellular Physiology | 12 | 1.628 |
| England |  | | |
| 1 | Circulation Research | 15 | 9.375 |
| 2 | Arteriosclerosis Thrombosis and Vascular Biology | 13 | 8.125 |
| 3 | Cardiovascular Research | 8 | 5 |
| 4 | Circulation | 5 | 3.125 |
| 5 | Journal of Biological Chemistry | 4 | 2.5 |
| 6 | Journal of Molecular and Cellular Cardiology | 4 | 2.5 |
| 7 | Journal of Physiology London | 4 | 2.5 |
| 8 | Scientific Reports | 4 | 2.5 |
| 9 | Cells | 3 | 1.875 |
| 10 | Frontiers In Cell and Developmental Biology | 3 | 1.875 |
